# Can household water sharing advance water security? An integrative review of water entitlements and entitlement failures

**DOI:** 10.1088/1748-9326/ad9851

**Published:** 2024-12-17

**Authors:** Melissa Beresford, Ellis Adams, Jessica Budds, Leila M Harris, Wendy Jepson, Tennille Marley, Chloe Nguyen, Amber Pearson, Anaís Delilah Roque, Asher Y Rosinger, Sameer H Shah, Matthew Stellbauer, Amber Wutich

**Affiliations:** 1 Department of Anthropology, San Jose State University, San Jose, CA, United States of America; 2 Keough School of Global Affairs, University of Notre Dame, Notre Dame, IN, United States of America; 3 Department of Geography, University of Bonn, Bonn, Germany; 4 Institute for Resources, Environment and Sustainability & Institute for Gender, Race, Sexuality and Social Justice, University of British Columbia, Vancouver, British Columbia, Canada; 5 Department of Anthropology and Development Studies, University of Johannesburg, Johannesburg, South Africa; 6 Department of Geography, Texas A&M University, College Station, TX, United States of America; 7 American Indian Studies, Arizona State University, Tempe, AZ, United States of America; 8 Charles Stewart Mott Department of Public Health, Michigan State University, East Lansing, MI, United States of America; 9 Nicholas School of the Environment, Duke University, Durham, NC, United States of America; 10 Department of Biobehavioral Health, Pennsylvania State University, State College, PA, United States of America; 11 Department of Anthropology, Pennsylvania State University, State College, PA, United States of America; 12 School of Environmental & Forest Sciences, University of Washington, Seattle, WA, United States of America; 13 Texas Water Resources Institute, College Station, TX, United States of America; 14 School of Human Evolution and Social Change, Arizona State University, Tempe, AZ, United States of America

**Keywords:** water insecurity, entitlement theory, informal water systems, social insurance, moral economies for water, water charity, water coping

## Abstract

An increasing number of studies find that water sharing—the non-market transfer of privately held water between households—is a ubiquitous informal practice around the world and a primary way that households respond to water insecurity. Yet, a key question about household water sharing remains: is water sharing a viable path that can help advance household water security? Or should water sharing be understood as a symptom of water *in*security in wait for more formalized solutions? Here, we address this question by applying Sen’s entitlement framework in an integrative review of empirical scholarship on household water sharing. Our review shows that when interhousehold water sharing is governed by established and well-functioning norms it can serve as a reliable transfer entitlement that bolsters household water security. However, when water sharing occurs outside of established norms (triggered by broader entitlement failures) it is often associated with significant emotional distress that may exacerbate conditions of water *in*security. These findings suggest that stable, norm-based water sharing arrangements may offer a viable, adaptive solution to households facing water insecurity. Nevertheless, more scholarship is needed to better understand when and how norm-based water transfer entitlements fail, the capacity of water sharing practices to evolve into lasting normative entitlements, and the impact of interhousehold water sharing on intrahousehold water security.

## 1. Introduction

Achieving global household water security is one of the greatest challenges facing humanity in the 21st century. Nearly 4.4 billion people worldwide lack access to safely managed drinking water and more than half the world anticipates harm from their current drinking water source (Greenwood *et al* 2024, Miller *et al* 2024). A key feature of this challenge is highlighted by scholars, activists, and practitioners who largely agree that household water security does not just mean ensuring that people have physical access to water, but rather that people have access to sufficient, safe, and reliable water for sustaining their health, livelihoods, and well-being (Jepson *et al* 2017, 2019, Sultana and Loftus 2019, Shah 2021). Understanding household water security this way shifts the framing from a purely technical or supply-side problem to a broader mandate for delivering water in ways that recognizes its role in facilitating human capabilities and flourishing (Jepson *et al* 2019).

Water scholars, policy makers, and activists agree on the importance of formalized access to safe and sufficient water (i.e. access that is legally codified and regulated) (Heller 2022). For instance, repairing and expanding piped networks, creating more equitable markets and state support systems, and innovating technologies to leap-frog access barriers are clearly integral to expanding water security worldwide. Yet, significant challenges—bureaucracy, corruption, economic and social exclusion, and market failures—undermine these formalized solutions and upend, disrupt, or delay getting people the water they need (Jepson 2012, Mirosa and Harris 2012, Stoler *et al* 2022, Wutich *et al* 2023, Thomson *et al* 2024a).

Many scholars have therefore highlighted the potential of *informal* water provisioning to complement formalized water delivery and management efforts (Whittington *et al* 1989, Solo 1999, Schwartz *et al* 2015, O’Donnell and Garrick 2019, Smiley 2020, Garrick *et al* 2023). Informal water provisioning encompasses water access and governance that are outside of state purview and control, such as informal water providers, grass-roots community organizations, and infrastructural tinkering (Whittington *et al* 1989, Collignon 1999, Peloso and Morinville 2014, Schwartz *et al* 2015, Wutich *et al* 2016, Garrick *et al* 2019, 2023, Adams *et al* 2020, Quimby *et al* 2023, Archidiacono *et al* 2024). Studies on informal water provisioning demonstrate that in the absence of reliable, safe, secure, and affordable formal water options, households and communities creatively devise their own solutions outside of state support and regulation. However, an important debate is the extent to which informal water provisioning can serve as a just, sustainable, and long-term pathway for achieving household water security (Schwartz *et al* 2015, Wutich *et al* 2023, Thomson *et al* 2024a).

**Figure 1.**
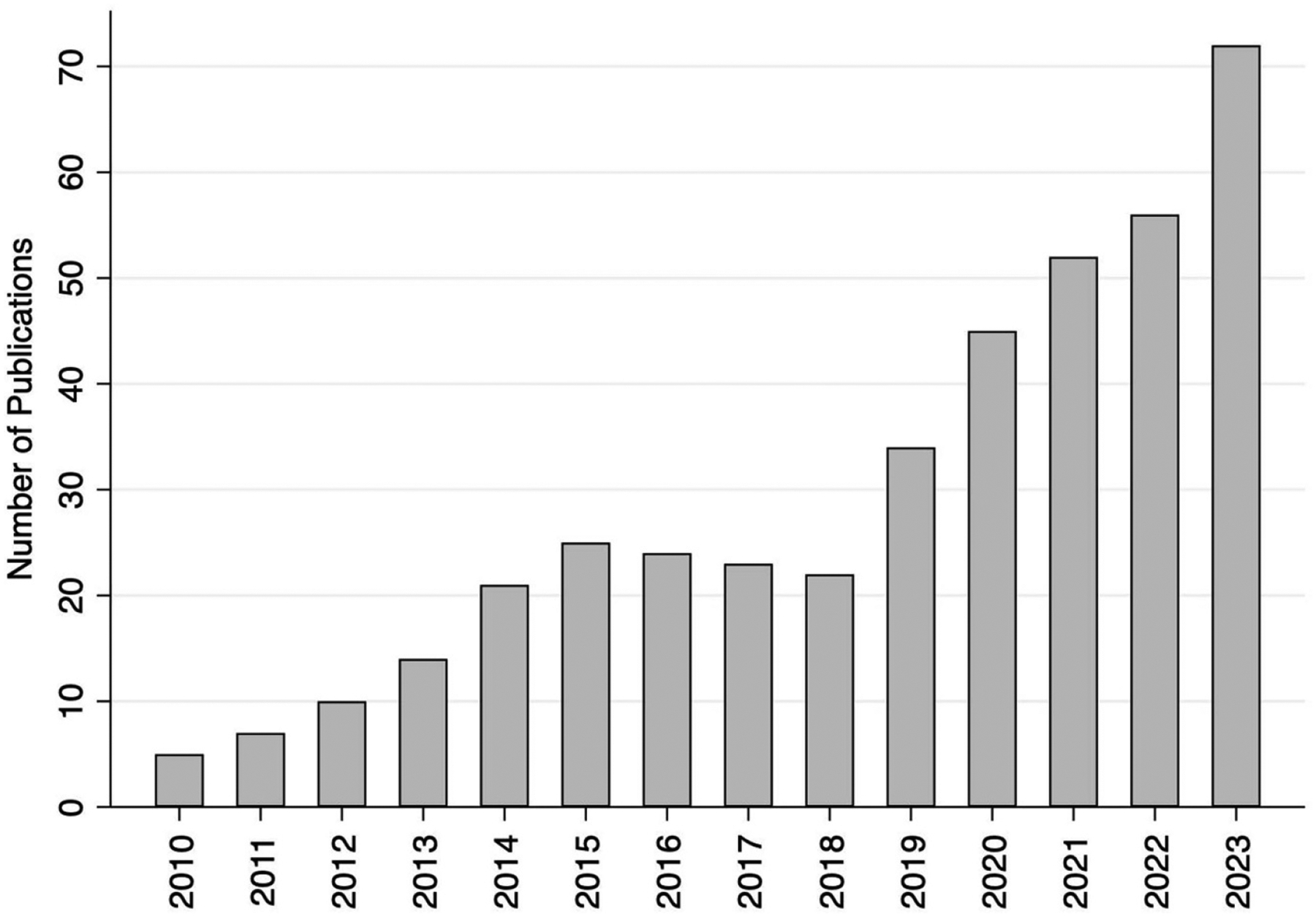
Household water sharing studies have increased substantially since foundational papers on the topic were published in 2010. Data: Scopus query on 10 May 2024 using search terms ‘household’ AND ‘water shar*.’ Studies from 2024 are not included in this figure due to incomplete data.

Household water sharing—the non-market direct transfer of privately held water between households (Wutich *et al* 2018, Brewis *et al* 2019)—is one form of informal water provisioning that has gained significant scholarly attention in recent years (figure 1)^15^. Common examples include: giving a bucket or jerry can of water to a neighbor; hauling water for those who lack strength or appropriate equipment; or allowing another household to use an on-property well or tap. Increasing ethnographic research and large-scale surveys demonstrate that these informal water exchanges between households are ubiquitous in water-insecure communities around the world and a key way that households meet their immediate water needs (Wutich *et al* 2018, 2022, Rosinger *et al* 2020).

But, as with the broader literature on informal water provisioning, a key question about household water sharing remains: Is water sharing a viable path that can help advance household water security? Or should water sharing be understood as a temporary measure, and thus a symptom of water *in*security in wait for more formalized solutions?

Here, we address this question by applying Sen’s (1981) entitlement framework to assess the peer-reviewed literature on household water sharing (beginning with the earliest widely known studies focused on the phenomenon). The entitlement framework provides a systematic way to identify the pathways through which households access water and assess whether or not those pathways help advance or undermine broader human capabilities (Sen 1988, 2005, Anand 2004, 2010, Wutich and Brewis 2014, Jepson *et al* 2017, 2019). We thus examine the entitlement arrangements that undergird water sharing practices reported in the literature to understand the conditions under which they may advance or undermine household water security. In doing so, we point to fruitful new directions for household water sharing and entitlement scholarship that can advance water (in)security research and help expand the next generation of water economics.

## 2. Background

### 2.1. Entitlement theory and water insecurity research

Entitlement theory, developed by Amartya Sen (1981) and recognized with the 1998 Nobel prize in economics, revolutionized the study of development and resource economics—demonstrating that food insecurity (at the household level) and famine (at the societal level) are not caused by a *lack* of food, but rather occur when people lose their ability to *access* food in legitimate or socially sanctioned ways. At the time, it marked a significant shift in how development economists understood food insecurity, moving research away from traditional metrics of per capita food supply to instead explore the distribution mechanisms that enable or constrain household food access (Webb *et al* 2006).

‘Entitlements’ refers to the legitimate, or socially sanctioned, pathways that a household uses to access a resource, such as water or food. The broad categories of entitlements that Sen originally outlined include direct production entitlements (e.g. owning land to grow food), trade entitlements (e.g. earning wages to buy food), labor entitlements (e.g. working for food), and transfer entitlement (e.g. being gifted food). Sen explained that households each possess a bundle of entitlements that they can use to secure essential resources. The more reliable and robust those entitlements are, the more likely it is that the household will be food (or water) secure. However, different entitlements may fail—due to direct means (e.g. drought destroys a household’s crops) or indirect means (e.g. job loss means that a household does not have money to buy food). When households have limited or unreliable entitlement options, or when too many of their entitlements fail at once, they experience heightened risk of food insecurity and starvation.

Sen’s analysis initially focused on societies with formalized property rights, where legal frameworks clearly defined entitlement pathways via rights to the ownership and the transfer of resources. For instance, theft and bribery are generally not considered entitlement pathways because they are not seen as legitimate or socially sanctioned in most societies (Sen 1981). However, subsequent research expanded the entitlement concept beyond Western legal understandings of property rights to include *informal* or normatively sanctioned means of resource access (Fine 1997, Leach *et al* 1999, Devereux 2001, Ribot and Peluso 2003, Sen 2008)^16^. In other words, scholars now recognize that entitlements are not always governed by formal legal systems but can also be understood as ‘legitimate’ paths for resource acquisition according to social norms and community-level institutions (Devereux 1996, Leach *et al* 1997, 1999, Sen 2008). For example, communal sharing, bartering, or other informal agreements are entitlement pathways that provide households resources on the basis of their normatively understood social membership (Devereux 2001, Johnson and Forsythe 2002, Johnson 2004).

Despite its prominence in food insecurity research, entitlement theory was not widely incorporated into water insecurity research until the 2000s. This early work on water entitlements drew inspiration from Sen’s scholarship to similarly challenge the idea that water insecurity should be measured solely by the total availability of water in a region. Like Sen, early water entitlement scholars demonstrated that water insecurity must instead be understood as households’ ability to reliably access safe and sufficient water in socially sanctioned ways (Anand 2004, 2010, Mehta 2007, 2014, Wutich and Brewis 2014, Dapaah and Harris 2017). Around this time, new bodies of scholarship emerged that sought to document and understand the diverse pathways that water insecure households rely on for water access, and how the failures of those pathways can result in water insecurity at various scales (table 1).

### 2.2. Household water sharing: a transfer entitlement and a response to broader entitlement failure

Water sharing became central to water insecurity research as scholars recognized it as a key experience of household water insecurity (Wutich *et al* 2018, Wutich and Beresford 2019, Venkataramanan *et al* 2020). In early theoretical research, water sharing was described as a form of reciprocity that is need-based and symbolically meaningful (Wutich 2011a, Zug and Graefe 2014, Schnegg and Linke 2015). Building on global empirical scholarship from anthropology, geography, and cognate fields, increasing water insecurity work has focused on a range of water sharing practices. This work has empirically demonstrated that: (1) water sharing is a ubiquitous practice in water insecure sites around the world; and (2) water sharing is a key informal practice that households rely upon for water.

As this scholarship grew, a distinct trend emerged: in some cases, water sharing operates as a clear transfer entitlement—one that is informally governed and sanctioned by social norms and community institutions. In such cases, norms for the direct transfer of water are well understood and predictably observed; individuals within the community possess implicit knowledge of the dos and don’ts for sharing water, including who can ask whom, how much water one can reasonably ask for, in what situations it is appropriate to ask for water, and in some cases, what is expected in return (e.g. Zug and Graefe 2014, El Didi and Corbera 2017, Schwieger *et al* 2022). In other documented cases, however, water sharing occurs outside of clearly understood social norms. In such cases where the norms of giving and receiving water are not well-established or well-understood—or may be different across ethnic, class, gender, racial categories, or other social divisions—people might ask for water in ways that violate others’ expectations of what is or is not appropriate (e.g. Sultana 2011, Wutich 2011b, Pearson *et al* 2015, Brewis *et al* 2021). In such cases, it may also be true that the act of asking for water is a last-ditch effort when all other water entitlements have failed. Water sharing in such contexts operates as an emergency response to entitlement failure rather than established transfer entitlement (table 2).

Although water sharing as a transfer entitlement and water sharing as a response to entitlement failure both result in the transfer of water (i.e. extending water access), scholars have debated whether or not the act of water sharing actually helps to advance household water security or if it instead serves as an indicator of broader water *in*security. Understanding that household water security encompasses not just water access, but access in ways that facilitate and support human capabilities, well-being, and flourishing, highlights the need to decipher when and how different conditions of water sharing (as an entitlement pathway vs. as a response to entitlement failure) may help advance or undermine water security goals.

In what follows, we present an integrative review of the peer-reviewed literature on household water sharing to understand the entitlement pathways that undergird water sharing arrangements and how these arrangements may enhance or threaten household water security. Our goal is to synthesize the existing scholarship on water sharing to outline new directions for future work on water sharing and water entitlement scholarship.

## 3. Approach

We employed established methods for conducting an integrative review (Torraco 2005, Whittemore and Knafl 2005, Cronin and George 2023) of household water sharing research from 2010 to present, beginning the year before the earliest papers dedicated to the phenomenon (Wutich 2011a, 2011b, Zug and Graefe 2014, Pearson *et al* 2015). Unlike systematic reviews, integrative reviews are designed to go beyond summarizing previous studies on a well-established topic of study; instead, they use inductive methods to identify major themes across studies from diverse fields and modes of scholarship (including qualitative and quantitative approaches) for the purpose of integrating findings to define new research directions (Torraco 2005, Cronin and George 2023). Integrative reviews are particularly useful for emerging or evolving areas of scholarship that span many subfields and are not yet well-integrated. Systematic reviews are generally not appropriate for newly developing fields (e.g. water sharing), or for reviews that aim to combine concepts (as in our case with combining water sharing and entitlement theory), or for reviewing studies that use a diverse array of qualitative and quantitative methods (Haddaway *et al* 2020, Uttley *et al* 2023).

For our integrative review, we identified water sharing studies by searching both Scopus and the web of science for the full text of peer-reviewed research articles (in English) using the search terms ‘household’ AND ‘water shar*’. Our search returned 450 results on Scopus and 29 results on web of science. We removed duplicates and publications that cite other studies on household water sharing but do not investigate inter-household water sharing practices directly. After this step, our remaining sample consisted of 86 papers for screening. We read the full text of all 86 papers; 47 analyzed empirical examples of inter-household water sharing—the direct transfer of water from one household to another. Thirty-nine papers focused on other forms of sharing that facilitated water acquisition (e.g. sharing information or equipment). These papers that focused on other forms of sharing were excluded from our analysis because they do not constitute the direct transfer of privately held water between households.

Using Sen’s theoretical framework (see table 2), two authors re-reviewed each of the remaining 47 publications to identify the cases of water sharing that occurred as part of a larger entitlement system vs. occurred outside of normative practices as a coping response to failures in production, trade, labor, exchange, or multiple and cross-cutting entitlement failures. We then used established techniques for inductive theme analysis (Ryan and Bernard 2003) to determine (a) how entitlement pathways operate in water sharing arrangements (both as entitlement *and* as a response to entitlement failure); and (b) how these water sharing arrangements enhance or detract from household water security. Below we outline these results before synthesizing the two bodies of work in our discussion.

## 4. Water sharing as a transfer entitlement

When water sharing occurs in a socially sanctioned way, guided by understood norms for the transfer of water, it constitutes a transfer entitlement. These norm-based water sharing systems largely fall into three categories: informal *social insurance systems, moral economies for water*, and *water charity* (table 3). Each of these norm-based systems consists of distinct features that shape when and how households receive water transfers and under what conditions they bolster or undermine household water security.

### 4.1. Social insurance systems

Early research on household water sharing networks described them as *social insurance systems* (Wutich 2011a, 2011b, Pearson *et al* 2015, Schnegg and Linke 2015), and this conceptualization is still common (e.g. Bukachi *et al* 2021, Ford *et al* 2022). In a social insurance system, households mitigate risk by pooling resources and/or having established norms for the redistribution of resources during times of need (Schweizer *et al* 1998). Studies on water sharing as a form of social insurance demonstrate that households around the world intentionally cultivate water sharing arrangements as a transfer entitlement pathway because they know and expect that their primary production and trade entitlements for water will (on occasion) fail (Pearson *et al* 2015, Schnegg and Linke 2015, Drew and Rai 2016, 2018, Schnegg 2016, Bukachi *et al* 2021).

Key examples of water sharing as a form of social insurance include studies in arid regions of Northwestern America and Sub-Saharan Africa in which households construct broad sharing networks via the proactive transfer of many types of resources (e.g. giving gifts and volunteering labor), expecting they will need to rely on these networks during drought and other seasonal water shortages (Pearson *et al* 2015, Schnegg 2015, Schnegg and Linke 2015, Ford *et al* 2022, Lerback *et al* 2022, Ilboudo Nébié *et al* 2024). For instance, in rural mountain communities of Baja California Sur in Mexico, neighbors regularly offer and engage in labor assistance on each other’s ranches to develop social and reciprocal relationships, in part, because they expect to ask for water from their neighbors when their wells fail due to interannual water shortages (Lerback *et al* 2022).

Well-functioning social insurance systems primarily occur among in-group relations (often ethnic or kinship structures)^17^. For instance, Pearson *et al*’s (2015) work in Uganda found that strongly embedded social norms among Bahima and Bairu agropastoral communities facilitated water sharing networks that households relied upon during the dry season when their primary sources of water were not available; ethnic outsiders living in proximity were excluded from these exchange networks and thus more susceptible to water insecurity. Later studies illustrate that water sharing in social insurance systems does not occur indiscriminately, but rather tracks with established group relations that are typically ordered by social distance (Schnegg 2015, Drew and Rai 2016, Eichelberger 2018, Bukachi *et al* 2021). In other words, norms often dictate who is obligated to share water with whom based on how socially close they are to one another. Most studies indicate that households cultivate these norms to mitigate expected water shortages driven by climate variability or water systems failures (Brewis *et al* 2019, Rosinger *et al* 2020, Jepson *et al* 2021, Ford *et al* 2022, Lerback *et al* 2022). However, a small number of recent studies show that households also cultivate and rely on water sharing norms to alleviate the time and labor burdens of fetching and transporting water (Eichelberger 2018, Ford *et al* 2022, Ilboudo Nébié *et al* 2024).

### 4.2. Moral economies for water

Some scholars note, though, that the emphasis on risk aversion as a motivating factor for social insurance systems does not adequately explain the diverse circumstances under which water sharing networks develop and persist. For example, risk aversion does not explain why wealthy households with sufficient resources who rarely run out of water participate in these networks—or how power dynamics operate in general within sharing systems—potentially leading to a misperception that social insurance systems are egalitarian networks that operate purely for risk redistribution (Beresford *et al* 2023).

Research on *moral economies for water* demonstrates that inequality and social stratification (in part) often drive the development of norms for water sharing (Wutich 2011a, 2011b, Zug and Graefe 2014, Gomez-Temesio 2016, El Didi and Corbera 2017, Schwieger *et al* 2022, Beresford *et al* 2023, 2024). In moral economies, shared cultural understandings of how justice, fairness, and social obligation should operate within a socially stratified community instigate a set of norms for the redistribution of water; these norms are kept in place by the threat of social sanctions for non-compliance (Beresford *et al* 2023). For instance, community norms may dictate that wealthier or more resource secure households have an obligation to provide for those in need (Zug and Graefe 2014, Gomez-Temesio 2016, El Didi and Corbera 2017). Households therefore often intentionally instigate water sharing relationships with others who are wealthier and more resource secure (Wutich 2011a, 2011b, El Didi and Corbera 2017, Schwieger *et al* 2022). The threat of gossip, snubbing, or social conflict (rather than risk aversion or altruism alone) often motivates households who have sufficient water to share with those without (Beresford *et al* 2023). But in moral economies, wealthier or more resource-secure households are also driven to share water to reinforce their social status and prestige and/or generate patronage benefits (El Didi and Corbera 2017, Schwieger *et al* 2022).

Unlike social insurance systems, moral economies for water are often relied upon long-term and may even be a household’s primary source of water (El Didi and Corbera 2017, Alves 2022). Similar to social insurance systems, moral economies typically function well in small, culturally homogenous communities (Beresford *et al* 2024). Scholarship has yet to explore how well moral economies function at larger scales or within culturally diverse populations.

### 4.3. Water charity

The water sharing literature also documents *water charity* as an important transfer entitlement. Charity must be understood as conceptually different from social insurance systems and moral economies because charity transfers occur unidirectionally without established or lasting social relationships or normative expectations of direct reciprocity (Milinski *et al* 2002, Pickles 2020, Parsell and Clarke 2022). Nonetheless, when water charity is established and predictable, it constitutes an important entitlement for many households. For instance, nascent work on water and homelessness in high-income countries indicates that households experiencing homelessness rely mostly upon purchasing bottled water (Meehan *et al* 2023), but regularly turn to charitable donations of water by local organizations, including shelters and mobile vans handing out bottled water (DeMyers *et al* 2017, Avelar Portillo *et al* 2023, Meehan *et al* 2023).

It is important to note that in charity systems no established relationships (or mechanism of social pressure) exist between givers and receivers. Additionally, in charity systems, the status difference between givers and receivers is typically large; engagement in the charity transfer often generates further status accrual for givers and status loss for receivers (Milinski *et al* 2002, Parsell and Clarke 2022). Social insurance systems and moral economies, on the other hand, may be driven (in part) by status and prestige accrual for givers, but they only generate status loss for receivers in limited circumstances. Research on water charity remains limited, and more studies are needed to understand if water charity, specifically, may trigger status loss, feelings of shame, or emotional distress for those who receive it.

### 4.4. Summary

Collectively, studies on water sharing in social insurance systems and moral economies for water show that well-functioning norm-based water sharing networks (i.e. those in which norms are regularly and predictably followed) are a key household entitlement that bolsters household water security. By engaging in norm-based water sharing systems households buffer themselves against water insecurity risks and strengthen social relationships with other households, thus leading to expectations that water sharing requests will be granted when needed. Some research has empirically explored the psychosocial or emotional effects of water sharing systems that appear to be well-functioning and norm-based, at least for some participants (Brewis *et al* 2021, Ford *et al* 2022); findings align with the broader anthropological literature on reciprocity that indicates water sharing under these circumstances should enhance positive emotion and well-being (Wutich *et al* 2022). Research on water charity, however, continues to be limited. Despite charity being a socially sanctioned pathway for households to receive water, more studies are needed to understand if (and under what circumstances) water charity, or other sharing practices, may trigger anxiety, status loss, feelings of shame, or emotional distress for those who receive it. In such circumstances, water charity may fundamentally undermine water security goals.

## 5. Water sharing and entitlement failure

The literature demonstrates that water sharing also occurs outside of established normative transfer systems (table 4). In most cases, sharing requests occur as a last ditch effort for a household to obtain water when all their other entitlement pathways have failed. But the water sharing literature also documents two other forms of non-normative water sharing: water sharing triggered by disaster response and water sharing as a form of political resistance.

### 5.1. Sharing as an emergency response to entitlement failure

Production entitlements for water (e.g. water ownership rights that provide water via wells, boreholes, springs, streams, and rainwater harvesting) often fail due to drought, contamination, or infrastructural breakdown (Sultana 2011, Rosinger *et al* 2020, Jepson *et al* 2021, Ford *et al* 2022). In these circumstances (or in the absence of available production entitlements for water), households rely on alternative entitlement pathways, including purchasing water from stores, kiosks, mobile vendors, or neighbors (i.e. trade entitlements [Wutich 2011a, Zuin *et al* 2011, 2014, Jepson and Brown 2014, Nganyanyuka *et al* 2014, Jepson *et al* 2021]) or work-for-water arrangements in which people perform labor (including sex work) for water (i.e. own labor entitlements (Sultana 2011, Zuin *et al* 2011, 2014, Nganyanyuka *et al* 2014, Owoola *et al* 2019, Alba *et al* 2022, Alves 2022, Nunbogu and Elliott 2022, Merkle *et al* 2023). However, vendors may be unreliable (Wutich *et al* 2016, Dapaah and Harris 2017), households may not have enough money to purchase water (Alba *et al* 2022, Alves 2022), and people may refuse resale arrangements or work-for-water arrangements without notice (Sultana 2011, Alba *et al* 2022). When these entitlement pathways fail and households do not have established norms and networks for sharing water (or their established sharing networks fail [i.e. transfer entitlement failure]), they may ask (or even beg) for water from others despite there not being a normative precedent for doing so (Sultana 2011, Collins *et al* 2018, Velzeboer *et al* 2018, Achore *et al* 2020, Jepson *et al* 2021, Alba *et al* 2022, Alves 2022, Wutich *et al* 2022).

Early ethnographic studies on water sharing illustrated these processes, and demonstrated that the act of asking (and being asked) for water outside or in violation of established norms causes significant amounts of emotional distress (Wutich and Ragsdale 2008, Sultana 2011, Wutich 2011a, 2011b). More recently, a series of larger-scale studies on water sharing support the links between water sharing as a coping mechanism and emotional distress: Rosinger *et al*’s (2020) study of almost 5500 households across 21 sites in 19 low- and middle-income countries demonstrated that water sharing is a ubiquitous practice around the world. Water sharing was practiced in all 21 sites surveyed, with 44.7% of households engaging in water sharing at least once within the previous month, and water systems failures were significantly associated with higher odds of water borrowing. A follow-up analysis of the same dataset by Wutich *et al* (2022), however, demonstrated that giving *and* receiving water were consistently associated with greater odds of reporting shame, upset, anger, and conflict over water, indicating that water sharing is a largely distressing practice for many people.

Recent scholarship suggests that water sharing may be distressing when it is unpredictable, especially if it occurs outside of an established system of norms for exchange (Wutich *et al* 2022, Beresford *et al* 2024). Three independent single-site studies on water sharing in Brazil (Jepson *et al* 2021), Ethiopia (Brewis *et al* 2021), and Kenya (Ford *et al* 2022) lend support to this theory: in all three studies, households that borrowed water from other households *infrequently* were more likely to report higher levels of emotional distress, but households that borrowed water often reported significantly less emotional distress. In each case, authors noted that more frequent water borrowing was likely an indication of established water sharing networks, while less frequent water borrowing was likely to comprise instances of asking for water outside of established social systems for exchange. Taken together, this body of work demonstrates that water sharing that occurs outside of established norms, as a last-ditch response to entitlement failure, likely *exacerbates* the experience of water insecurity despite providing emergency water access.

### 5.2. Water sharing in response to disaster

Disasters generally entail cascading entitlement failures for an entire population at once. Many communities who are prone to disaster develop broad norm-based sharing networks, in part, because they know they will need to depend on these networks when disaster occurs (Jones and Faas 2016, 2022). However, scholars have not yet documented whether and how these established norms for the transfer of other resources may facilitate water transfers, specifically, during post-disaster recovery. Yet, we do know that households that do not have established resource sharing networks turn to water sharing in the wake of disaster (Roque *et al* 2021, 2023a, Ito *et al* 2023, Jankovich-Rankovich *et al* 2024). Disaster response, in general, is typically characterized by an initial period of high cooperation cutting across social groups (Drury *et al* 2013, Nogami 2018), thus predicting that water sharing outside of established norms may occur widely and without associated status loss or stigma. However, high levels of cooperation after disaster typically decline as initial disaster response gives way to longer-term coping and reconstruction (Drury *et al* 2013, Nogami 2018). In this case, households that continue to depend on water sharing outside of normative transfer arrangements may contend with uncertainty, stigma, or shame. This is at least partially confirmed by Roque *et al*’s study of water sharing in Puerto Rico after Hurricane María (2021, 2023a) and Ito *et al*’s (2023) study of water sharing following the Gorkha earthquake in Nepal. But additional research on water sharing in the wake of disaster is needed to better understand the long-term impacts of water sharing on household water security in the wake of disaster (Jankovic-Rankovic *et al* 2024).

### 5.3. Water sharing as political resistance

A small number of studies have also documented water sharing as a form of political resistance. In these cases, water sharing generally occurs in response to large-scale entitlement failures that are triggered by state actions (Harris *et al* 2020). For example, recent work by Alves (2022) in Guinea-Bissau noted that households with a municipal water connection point regularly shared water with neighboring households without one. These sharing arrangements came to an abrupt halt when the government instituted water meters and began to charge residents for water per unit (rather than a flat access rate). Although many households stopped sharing water with their neighbors because their water bills increased, a small number of households continued to share water indiscriminately—despite increased bills—as a form of political resistance, noting that government attempts to charge citizens more for water were unethical. In another documented case, immigration and human rights activists along the U.S.–Mexico border leave water bottles and jugs to assist undocumented people crossing the border through the Sonoran Desert (Harris *et al* 2020). These cases show the need for more research on how the state disruption of entitlement systems might spur political action (Mitlin 2013, Harris *et al* 2020), and the extent to which water sharing as a form of state resistance impacts water security.

### 5.4. Summary

Water sharing widely occurs outside of established norms for the transfer of water. The literature indicates that these transfers typically operate as a type of emergency and last-ditch response to broader entitlement failures. In these circumstances, norms for the transfer of water are either not established or are violated. Taken together, the literature indicates that water sharing under these circumstances may allow households to obtain water to meet their most immediate needs, but doing so is associated with significant mental and emotional distress that ultimately exacerbates experiences of household water insecurity. Water sharing also occurs outside of established norms during the wake of disaster and as a form of political resistance. The literature in these two areas is not yet advanced enough to understand how these forms of water sharing may impact household water security.

## 6. Discussion

We set out to understand: can water sharing—as an informal practice—help advance household water security? Or should water sharing be understood as a *symptom* of water insecurity in wait for more formalized solutions? Our review of the literature strongly indicates that access to stable and well-functioning norm-based water sharing networks (i.e. social insurance systems and moral economies for water) has the potential to alleviate water burdens on households, especially in varying and unpredictable water environments. These norm-based institutions serve as key entitlement pathways that bolster household water security—knowing that one can fall back on their neighbors and/or community members in times of need can play a crucial role in alleviating burdens of worry and distress around water. Additionally, the literature indicates that norm-based water sharing systems have the potential to be dynamic and responsive to social and environmental conditions in ways that formalized state programs may not (e.g. Daapah and Harris 2017, Roque *et al* 2021, Ito *et al* 2023).

We emphasize, however, that we do not advocate for devolving responsibility for water security onto marginalized communities, and we acknowledge that there is abundant danger in viewing water sharing as a panacea or ‘silver bullet’ solution. All entitlement pathways have the potential to fail, and norm-based water sharing systems are no exception (Beresford *et al* 2023). But despite this potential, it is important to recognize that the independence and autonomy from state institutions that norm-based water sharing systems can provide may be crucial in regions where governments are absent, weak, corrupt, or not fully representative. In many instances, communities face water insecurity because they are marginalized and/or neglected by the state (Meehan 2014, Roque *et al* 2023b) or live in untrustworthy state regimes (Wilson *et al* 2023). In failed states or failed markets, water sharing has the potential to provide a viable and crucial alternative to reliance on ineffective or exploitative political and economic systems.

Nevertheless, our review also shows that water sharing often does not function in this way. Water sharing frequently occurs *outside* of norm-based transfer systems—as a last resort option to which people are forced to turn when all other entitlement pathways have failed. When water sharing is not a normative and socially sanctioned transfer entitlement it also has the potential to be associated with significant psychosocial and emotional costs. It might result in anxiety and distress, as individuals and households grapple with uncertainty about their water supply. The psychological burden of not knowing whether one’s basic needs will be met cannot be overstated, as it impacts on daily living and long-term planning (Sultana 2011, Workman and Ureksoy 2017, Rosinger 2023, Thomson *et al* 2024b). Under such circumstances, water sharing may instead be considered a symptom of water *in*security.

## 7. Unanswered questions and new research directions

Our review raises several important questions that the water sharing and water entitlements scholarship do not yet explicitly address:

### 7.1. When and why do normative transfer entitlements for water fail?

All entitlements have the potential to fail, including norm-based water transfers. The existing scholarship does not yet systematically examine *when* and *why* normative systems for transfer entitlements may not function well (or might fall apart), but the ethnographic literature gives some clues. First, it is clear that successful norm-based transfer entitlements require adequate resources to redistribute within the community (Scott 1977, Dirks *et al* 1980). Economic, political, or ecological crises that trigger extreme levels of water scarcity across an entire community (including abdication of state responsibility for water provisioning) mean that there may simply not be enough water to share, thus causing established norms for water sharing to collapse (Wutich 2011b, Alves 2022, Beresford *et al* 2023, 2024, Hamidi *et al* 2024). For example, during his research in northern Kenya, Rosinger (field notes, July 2024) noted that households hid water during a prolonged drought in northern Kenya so that when neighbors asked for water they could lie and say they had no water to avoid sharing. Another explanation is that as communities become larger and more diverse, and social distance between households increases, the increased formalization of governance systems pushes households to rely more heavily on formal or state-sponsored entitlement pathways (Schnegg 2015, Schwieger *et al* 2022) and potentially pull back from norm-based transfer systems. A third potential explanation is that increased migration in and out of communities may lead to greater social distance and weakened social bonds that disrupt the potential for norm-based social systems to become established (Wutich 2011a, Pearson *et al* 2015). Future scholarship on water sharing and entitlements should examine the political, economic, and ecological conditions that facilitate successful norm-based transfer entitlements to better understand the conditions that enable such systems to meaningfully advance household water security.

### 7.2. Can water sharing practices that begin as an emergency response to entitlement failure evolve into normative transfer entitlements?

The existing literature on water sharing, and the broader literature on entitlement theory, does not adequately address the dynamism of entitlement systems—especially how norm-based entitlements emerge. It remains unclear if and how water sharing that begins as an emergency coping response may evolve over time and potentially transition to becoming normative and a part of a newly recalibrated entitlement regime. The water sharing literature, however, does give some starting points. Social cohesion and social distance, distribution of resources, social status differentiation, underlying symbolic or value systems, and levels of scarcity all potentially impact a community’s ability to transition from sporadic water sharing to a more predictable and reliable entitlement system. Communities with strong social bonds and embedded norms for the redistribution and transfer of other resources are likely more capable of making this transition. Conversely, high levels of social distance, community transience, or mobility likely hinder the establishment of stable water sharing systems. Moving forward, understanding the factors that influence how water sharing can potentially transition from an emergency response to entitlement failure to a norm-based transfer entitlement is crucial for supporting communities facing water insecurity and building sustainable systems for the future.

### 7.3. How does water sharing impact intrahousehold water security?

Entitlement theory, at large, is often critiqued for its lack of consideration for intrahousehold dynamics and impacts (Devereux 2001). To date, the vast majority of water sharing literature focuses on the household as the unit of analysis, with data collection generally occurring with the head-of-household or person who is in charge of water for the household (Wutich *et al* 2017). However, water insecurity scholars are increasingly examining the intrahousehold dynamics of water provisioning decisions (e.g. Wutich 2009, Tsai *et al* 2016, Maxfield 2020, Tallman *et al* 2023, Hillesland and Doss 2024). This scholarship has not explicitly examined how different entitlement pathways (or different forms of entitlement failure) impact intrahousehold dynamics, as well as differentiated household members (with respect to age, social position, etc.). Moving forward, understanding the intrahousehold impacts of water sharing as a transfer entitlement vs. water sharing as a response to entitlement failure will enable researchers to gain a more holistic understanding of how water sharing can facilitate (or undermine) water security goals.

## 8. Conclusion

In short, the answer to whether household water sharing can advance water security is that it depends—it depends on the structure of the entitlement pathways through which water is being shared. When water sharing operates successfully as a norm-based transfer entitlement—with established norms that create legitimate expectations of when and how one will receive water in return—it has the potential to enhance household water security. However, when there is a lack of established norms for the transfer of water (i.e. requests for water are last-ditch emergency responses) water sharing may simply add to the experiential burden of water insecurity. More attention to these varying entitlements and entitlement failures will help scholars better understand if and how the diverse formal and informal ways that people access water ultimately help to enhance vs. undermine household water security.

## Figures and Tables

**Table 1.** Sen’s (1981) original entitlement categories as applied to water.

Entitlement category	Description	Key examples in the literature
Production	Own the means to water (e.g. property rights to groundwater or surface water).	Lerback *et al* (2022), Jepson *et al* (2021)
Trade	Buying or trading for water.	Zuin *et al* (2011, 2014), Jepson and Brown (2014), Wutich *et al* (2016), Dakyaga *et al* (2023, 2024), Garrick *et al* (2023)
Own Labor	Working for water.	Sultana (2011), Owoola *et al* (2019), Nunbogu and Elliott (2022), Alba *et al* (2022), Merkle *et al* (2023)
Transfer	Social systems (formal or informal) that provide the direct transfer of water	Wutich (2011a), Wutich (2011b), Zug and Graefe (2014), Pearson *et al* (2015), El Didi and Corbera (2017), Yates and Harris (2018), Wutich *et al* (2018), Schwieger *et al* (2022), Ford *et al* (2022)

**Table 2.** Analytical categories of water sharing according to Sen’s entitlement framework.

	Definition	Supporting citations
Water sharing as transfer entitlement	Social system with established norms for the direct transfer of water. Transfers are socially sanctioned via social membership.	Sen (1981), Fine (1997), Leach *et al* (1999), Devereux (2001)
Water sharing as response to entitlement failure	Water transfers that occur outside of an established normative social system (e.g. norms for the transfer of water between households are not established), and no normative social obligation exists between the giver and receiver.	

**Table 3.** Examples of water sharing as a transfer entitlement.

Water sharing as transfer entitlement	Description	Key citations
Social insurance systems	Households invest in social relationships and reciprocal exchanges (i.e. water, food, and other resources) with the understanding that they can reliably draw upon those relationships when they need water.	Pearson *et al* (2015), Schnegg and Linke (2015), Schnegg (2016), Drew and Rai (2016, 2018), Eichelberger (2018), Bukachi *et al* (2021), Brewis *et al* (2021), Lerback *et al* (2022), Ford *et al* (2022), Ilboudo Nebie *et al* (2024)
Moral economies for water	Cultural understandings of how justice and social obligation should operate in socially stratified communities facilitate the transfer of water between households. The threat of social sanctions and the potential for status accrual ensure that water transfers are predictable.	Zug and Graefe (2014), Gomez-Temesio (2016), El Didi and Corbera (2017), Schwieger *et al* (2022), Beresford *et al* (2023, 2024)
Water charity	One-way transfers of water between a benefactor and receiver with no expectation of reciprocity or mutual obligation.	Smiley (2016), DeMyers *et al* (2017), Avelar Portillo *et al* (2023)

**Table 4.** Examples of water sharing as an emergency response to entitlement failures.

Entitlement type	Entitlement failure	Water sharing emergency response	Key citations
Production	Water system failures (e.g. well breaks, runs dry, and becomes contaminated); drought causes failure of private rainwater harvesting system.	Households turn to neighbors and nearby acquaintances to ask for water, but responses can be unpredictable	Sultana (2011), Rosinger *et al* (2020), Brewis *et al* (2021), Jepson *et al* (2021), Ford *et al* (2022)
Trade	No money to buy water/pay water bill(s); no purchasing points available or vendors refuse to sell water.	Households ask for water from neighbors and acquaintances to tide them over, but requests are frequently refused	Wutich (2011a, 2011b), Sultana (2011), Jepson and Brown (2014), Nganyanyuka *et al* (2014), Wutich *et al* (2016), Velzeboer *et al* (2018), Brewis *et al* (2019), Wutich *et al* (2022)
Own-labor	Work-for-water arrangements (e.g. performing labor arrangements in exchange for water) are broken.	Households beg for smaller amounts of water for free, or turn to asking others they have never asked before for water in the interim	Sultana (2011), Alba *et al* (2022)
Transfer	Commodification and/or privatization of water (e.g. implementation of water meters or pricing changes for water) cause households to withdraw from existing water sharing arrangements.	Households turn to asking for water outside/beyond their previously established sharing network—e.g. asking for water from strangers or socially distant people	Jepson and Brown 2014; Alves 2022
Cascading entitlement failures	Disaster or state actions disrupt multiple entitlement pathways simultaneously.	Household share water with one another as emergency self-provision, especially in the absence or failure of NGO or government recovery efforts; households share water with other households as a form of political resistance	Roque *et al* (2021, 2023a), Ito *et al* (2023), Harris *et al* (2020), Alves (2022)

## Data Availability

All data that support the findings of this study are included within the article (and any supplementary files).

## Footnotes

15 A household is a group of individuals who live together and make joint decisions regarding consumption and production (Rappaport 2000, Netting *et al* 2023).

16 See Ribot and Peluso (2003) for a detailed explanation on debates around rights vs. access in property regimes.

17 For an operationalized definition of human groups (including ethnic groups), and explanation of human group behavior, see Moya (2023).
